# Seronegative Autoimmune Hepatitis A Clinically Challenging Difficult Diagnosis

**DOI:** 10.1155/2017/3516234

**Published:** 2017-07-06

**Authors:** Jagannath M. Sherigar, Arefiev Yavgeniy, Debra Guss, Nhu Ngo, Smruti Mohanty

**Affiliations:** New York Presbyterian Brooklyn Methodist Hospital, 506 6th Street, Brooklyn, NY 11215, USA

## Abstract

Autoimmune hepatitis (AIH) is a complex liver disease of unknown cause which results in immune-mediated liver injury with varied clinical presentations. Seronegative AIH follows a similar course to autoantibody-positive disease and diagnosis may be challenging. There are no single serologic tests of sufficient diagnostic specificity, and delay in appropriate treatment may lead to progression of the liver disease and liver failure. The revised conventional diagnostic criteria (RDC) scoring for AIH is complex and not routinely used in the clinical practice. The more recent simplified diagnostic criteria (SDC) scoring proposed by International Autoimmune Hepatitis Group in 2008 has wider application in routine practice facilitating the diagnosis of AIH with a specificity and sensitivity of ~90%. In this report, we describe a case of seronegative autoimmune hepatitis diagnosed using RDC. SDC score calculated in our case was 4 and was not diagnostic for AIH. We subsequently used the complex revised diagnostic criteria for definitive diagnosis. Some of the patients previously diagnosed as cryptogenic active hepatitis of unknown etiology probably had an unrecognized diagnosis of seronegative autoimmune hepatitis. SDC scoring may not be applicable in patients with seronegative autoimmune hepatitis. These patients should be reassessed by using RDC.

## 1. Introduction

Autoimmune hepatitis (AIH) is a complex chronic autoimmune liver disease of unknown etiology that occurs as one's immune system attacks liver cells and creates a chronic inflammatory state which may not only progress to cirrhosis but also could cause acute liver failure. AIH occurs in children and adults of all ages and all ethnicities, though it is more common in females between the ages of 40 and 50s. In the US, an annual incidence of 1 per 200,000 cases of AIH was reported [1]. Hallmark features of AIH include hyperglobulinemia, circulating autoantibodies, and histological features consistent with the chronic necroinflammatory state [2].

Liver pathology is not pathognomonic but it is essential for the diagnosis in cases with negative serologies. In 1999, International Association of Autoimmune Hepatitis Group (IAIHG) proposed revised diagnostic criteria (RDC) that were complex and meant for scientific purposes [3]. On the other hand, in 2008, IAIHG proposed simplified diagnostic criteria (SDC) as a diagnostic tool in individual cases of AIH to help guide treatment [4]. In this report, we described a case of seronegative AIH diagnosed with RDC that could not meet scoring criteria of SDC to diagnose seronegative AIH.

## 2. Case Report

58 y/o female with no medical history presented with the upper abdominal pain of one-day duration associated with nausea and vomiting. The patient was nonalcoholic and reportedly had no significant medical history. She was not taking any prescription or nonprescription medication. There was no recent travel history. Physical examination was unremarkable except epigastric and right upper quadrant tenderness. Initial laboratory studies were remarkable for elevated liver enzymes (ALT 1694 IU and AST 1139 IU) including alkaline phosphatase (ALP) 291 IU and total bilirubin of 5.9 (direct bilirubin 3.7). Ultrasonography and subsequent magnetic resonance cholangiopancreatography (MRCP) of abdomen showed numerous gallstones and normal common bile duct (CBD) without any intraductal stone. Viral serologies for hepatitis A, B, and C were negative. Conventional serologies for AIH were unremarkable with negative ANA, ASMA, anti-LKM 1 and 3 antibodies (staining by indirect immunofluorescence), and normal serum IgG level. On the other hand, perinuclear anti-neutrophil cytoplasmic antibody (p-ANCA) was only positive with level 1.3 (normal < 1.0). Additionally, EBV, CMV, and HSV IgM antibodies were normal.

**Figure 1:**
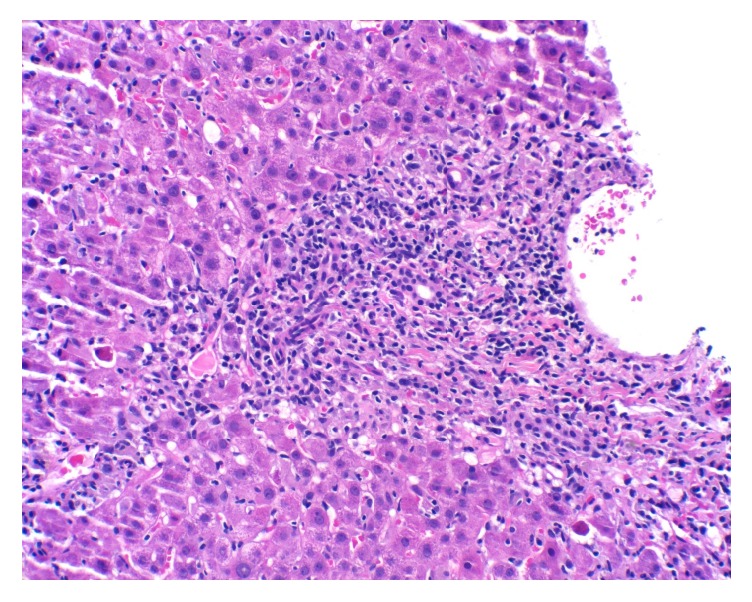
Interface hepatitis: portal tract with inflammatory infiltrate which extends past the limiting plate into the surrounding parenchyma (20x magnification).

**Figure 2:**
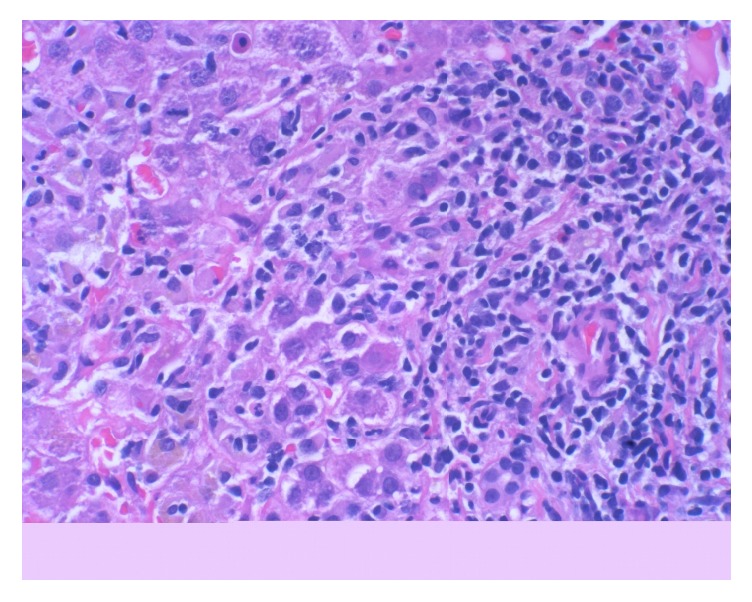
Hepatocytes at the interface are surrounded by inflammatory cells, causing hepatocellular damage (40x magnification).

The core liver biopsy showed portal tracts with moderate to severe mixed infiltrate of lymphocytes, plasma cells, and eosinophils, associated with severe grade 4 circumferential interface hepatitis (Figures 1 and 2). There was an extension of the inflammatory infiltrate deep into the parenchyma, with apoptotic and necrotic hepatocytes (Lobular activity 2) showing mild fibrosis (Stage 1). The bile ducts were preserved. Her AIH diagnostic score by using SDC was only 4 and was not sufficient to diagnose AIH. Unfortunately, this patient was not tested for SLA/LP-antibodies prior to treatment with steroids; however, even if she was positive, her SDC score would have been 6 and still was not meeting the criteria for definitive diagnosis of AIH. We used RDC scoring for a definite diagnosis of AIH (total score 17). Her liver enzymes improved dramatically just within two doses of steroid treatment (Table 1) with complete normalization of her liver enzymes in four weeks of a tapering dose of steroid treatment and introduction of azathioprine. Subsequently azathioprine was continued as maintenance of immunosuppression. Patient moved to different state after she was started on azathioprine and lost to follow-up.

## 3. Discussion

An immune response targeting liver autoantibodies are thought to initiate and perpetuate the damage [2]. Clinical manifestation includes a wide spectrum of presentations, from asymptomatic patients to those with considerable symptoms and occasionally with acute fulminant liver failure [5].

AIH type 1, also called classic AIH, is characterized by the presence of ANA and/or smooth muscle autoantibodies (ASMA). Type 2 AIH is defined by the presence of specific autoantibodies against liver and kidney microsomal antigens (anti-LKM type 1 or infrequently type 3) and or anti-liver cytosol type 1 antibody (ALC-1) [2]. Diagnosis is based up on characteristics serology and histology and exclusions of other forms of the chronic liver disease. Both ANA and ASMA present in about 70–80% of AIH cases [6]. If conventional autoantibodies are not detected, atypical perinuclear anti-neutrophilic cytoplasmic antibodies (p-ANCA) may be found. [2]. Antisoluble liver antigen/liver pancreas antigen (anti-SLP/LP) found both in type 1 and type 2 AIH may be helpful in diagnosing AIH without classical autoantibodies. Circulating autoantibodies are absent in about 10% AIH patients [7]. Patients with acute presentation (as in our case) autoantibodies and elevated IgG levels may be lacking and that autoantibodies may become positive only weeks or months later. Patients with seronegative autoimmune hepatitis (SAIH) have demographic, biochemical, and histologic features of classical AIH but negative autoimmune serology. They may be treated effectively as classical AIH with corticosteroids. As early as 1990, Czaja and colleagues in their retrospective analysis of chronic active hepatitis of cryptogenic nature and AIH with positive serology concluded that discrimination was not possible based on any individual feature except for the immunoserology markers. These two groups of patients had similar prognosis after corticosteroid therapy [8].

There is enough evidence now to suggest that some of the patients previously classified as cryptogenic chronic active hepatitis probably had AIH, but their serology was negative for conventional autoantibodies [8–10]. A cohort of 126 consecutive patients with presumed cryptogenic hepatitis referred to a university hospital were reanalyzed by Heringlake and colleagues. They found that only one-third of patients with initially presumed cryptogenic liver disease remained cryptogenic, while another third of patients were reidentified as seronegative autoimmune hepatitis by the IAIH-score with obvious benefit from immunosuppressive therapy [10]. An original scoring system initially developed by IAIHG in 1993 and subsequently revised in 1999 (RDC) was not designed for daily clinical practice as they were too complicated for bedside use. SDC scoring, based on serology, immunoglobulin level, liver histology, and exclusion of viral hepatitis, is widely used for diagnosis of individual patients. The SDC has been tested in several studies, and a reliable diagnosis of AIH can be made using a very simple diagnostic score with a high degree of sensitivity and specificity. The score was found to have 88% sensitivity and 97% specificity (cutoff > 6) and 81% sensitivity and 99% specificity (cutoff > 7) in the validation study [4]. Although SDC was recommended to diagnose individual cases of AIH in clinical practice, a total score of 4 was not enough to diagnose AIH in our case. Subsequently, for the definitive diagnosis, we used conventional RDC scoring. An acute case of seronegative AIH like our case was reported by Yilmaz et al., which had a calculated SDC score of 4, which was not sufficient to make the diagnosis. Diagnosis of probable AIH was made in their case per pretreatment RDC score of 14 [11]. SDC and RDC have very similar diagnostic accuracies for patients with typical features of AIH. Diagnostic criteria used for conventional AIH may not be applicable to cases with seronegative autoimmune hepatitis. Current report suggests that RDC is more helpful in diagnosing patients who are negative for conventional autoimmune serology.

## 4. Conclusion

Seronegative autoimmune hepatitis appears to have similar characteristics to conventional AIH and is treated with the same medications. Patients with probable or nondiagnostic SDC scores for AIH may be reassessed by using RDC scoring. RDC appears to be more accurate in the diagnosis of AIH in unclear cases like negative conventional autoimmune serologies.

## Figures and Tables

**Table 1:** Liver function studies before and after initiation of treatment with oral prednisone therapy.

Labs	Initial	Day before steroid therapy	Day 2 (steroid therapy)	Day 5	Day 7	3 weeks	4 weeks after therapy
Total bilirubin	5.9	9.4	7.3	4.3	2.7	1.1	0.3
Direct Bili	3.7	8.0	6.3	3.6	—	—	—
AST	1,139	877	349	184	130	25	9
ALT	1,694	1,328	916	599	456	97	21
ALP	291	201	197	155	167	112	75
INR	1.09	1.20	1.08	1.03	0.90	0.95	0.95
Alb	3.6	2.9	3	2.9	3.6	3.4	3.6

Bilirubin: mg/dl; AST, ALT, and ALP: unit/lit; Alb: mg/dl.
